# A cross-sectional study of environmental, dog, and human-related risk factors for positive canine leptospirosis PCR test results in the United States, 2009 to 2016

**DOI:** 10.1186/s12917-019-2148-6

**Published:** 2019-11-15

**Authors:** Amanda M. Smith, Andréia Gonçalves Arruda, Michelle D. Evason, J. S. Weese, Thomas E. Wittum, Donald Szlosek, Jason W. Stull

**Affiliations:** 1 Department of Veterinary Preventive Medicine, College of Veterinary Medicine, The Ohio State University, Columbus, OH 43210 USA; 2 Department of Companion Animals and Department of Health Management, Atlantic Veterinary College, University of Prince Edward Island, C1A4P3, Charlottetown, PE Canada; 3 Department of Pathobiology, Ontario Veterinary College, Centre for Public Health and Zoonoses, University of Guelph, N1G2W1, Guelph, Ontario Canada; 4 IDEXX Laboratories, Inc, Westbrook, ME 04092 USA

**Keywords:** Canine, Leptospirosis, Zoonosis, PCR testing

## Abstract

**Background:**

Canine leptospirosis is a reemerging zoonotic disease concern in North America, and a better understanding of its epidemiology is needed. Wide-scale use and subsequent analyses of polymerase chain reaction (PCR) testing may provide additional insight into leptospirosis. This study aimed to describe temporal trends, to descriptively map, and to identify environmental, dog, and human-level factors associated with positive canine leptospirosis PCR test results in the United States.

**Results:**

Data obtained from IDEXX Laboratories, Inc. on 40,118 canine leptospirosis PCR tests run in the United States between 2009 and 2016 were evaluated. Climate and socioeconomic (e.g. urban influence, income) data were obtained from public databases. Choropleth maps were created to identify high test-positive proportion areas and a cross-sectional analysis was completed with generalized (univariable, followed by multivariable) mixed logistic regression models accounting for county within state to identify significant predictors for a positive test. Overall test-positive proportion was 5.4% across the United States, with the regional point estimate highest in the southwest (8.1%). In the final multivariable model, the odds of a positive test were greater for male dogs (Odds Ratio [OR] = 1.28) and dogs 0–4 years of age (ORs ranged from 0.35–0.71 for the other age groups). The odds of a positive test were greater for dogs living in areas with wet environmental conditions (OR = 1.24). Season and temperature, as well as the interaction between them, were significant predictors of a positive test. Dogs had a greater probability of testing positive during cool temperatures (< 4 °C) compared to the other temperature categories in the fall season.

**Conclusions:**

These findings based on PCR testing allow for an improved understanding of factors influencing a positive canine leptospirosis PCR test and will assist targeted education and prevention efforts.

## Background

Leptospirosis is a worldwide bacterial disease that affects humans and animals and is regarded as re-emerging in dogs in North America. *Leptospira* spp. are tightly coiled spirochetes shed in urine and transmitted by maintenance hosts, such as rats, mice, and raccoons [1]. There are multiple pathogenic *Leptospira* spp. serovars circulating in the United States, each with different wild and domestic animal reservoir species and geographic spread [1]. Serovars identified in dogs in the United States include Canicola, Icterohaemorrhagiae, Autumnalis, Pomona, Grippotyphosa, and Bratislava [1]. Dogs acquire the bacteria from direct contact of mucous membranes or broken skin with urine of infected individuals or contact with food, water, or soil contaminated with infectious urine [2]. Leptospirosis can cause a wide spectrum of clinical illness in canines, ranging from subclinical to severe hepatic or renal failure, with the possibility of death [3].

Previous studies have identified spatial and temporal trends in the prevalence of canine leptospirosis cases in the United States [4–6]. Increased prevalence has commonly been identified from the west coast, the upper midwest, the northeast and the southeast [4, 5]. A seasonal increase in cases has been previously reported in late summer and fall [6, 7]. Additionally, environmental, dog owner, and dog-level risk factors have been linked to canine leptospirosis cases in previous studies [6, 8–13]. Despite these previous studies, the epidemiology of canine leptospirosis remains poorly defined, with studies generally limited to a single geographical area or a single subset of factors (e.g. environmental factors). The current study collectively examines factors for a positive polymerase chain reaction (PCR) test from many predictor subsets (temporal, geographical, environmental, animal) on a national scale to highlight risk factors and explore current canine leptospirosis epidemiology in the United States.

Reliance on serologic testing, with varying criteria for defining a positive test result, has contributed to the challenge of studying this bacterium in dogs. The microscopic agglutination test (MAT) has been widely used to diagnose leptospirosis in dogs, and previous canine risk factors and prevalence studies have used MAT data [3, 5, 12, 14]. In recent years, PCR leptospirosis testing has become increasingly used in clinical veterinary medicine and may reduce the interpretation challenges commonly encountered with MAT (e.g. a single acute MAT has been found to have lower sensitivity for predicting urine shedding compared to a urine PCR assay, is not as influenced by recent vaccination, and since it does not identify serovars, there are no concerns about cross reaction among serovars) [3, 15]. The PCR test detects both viable and non-viable *Leptospira* spp. nucleic acid and not the organism itself. Since it can detect non-viable nucleic acid, it may take extensive antimicrobial use for a false negative to occur [3]. Nationwide analysis of the spatial and temporal distribution of canine leptospirosis and evaluation of associated risk factors using PCR test data is important to provide additional insights into this complex disease. The objectives of this study were: (1) to describe temporal trends of canine leptospirosis PCR test results in the United States, (2) descriptively map canine leptospirosis PCR test results in the United States, and (3) investigate the association between environmental, seasonal, dog- and human-level factors, using a large dataset of canine leptospirosis PCR-test results.

## Results

Over the 8-year study period, a total of 41,370 test results were provided. Duplicates consisted of 1017 entries for which at least one exact matching entry (concordant test results) was present. The test entry with the most recent date for each dog was left in the dataset, resulting in the removal of 1152 test entries (range 1–116 duplicate entries removed per dog, median 1). Forty-one dogs had duplicate entries with discordant test outcomes. All entries for these dogs were removed from the data, resulting in the removal of 100 test entries (range 2–10 duplicate entries removed per dog, median 2). A total of 1252 duplicates were removed from the dataset due to duplicate entries, leaving 40,118 test results to be included in the analysis.

**Fig. 1:**
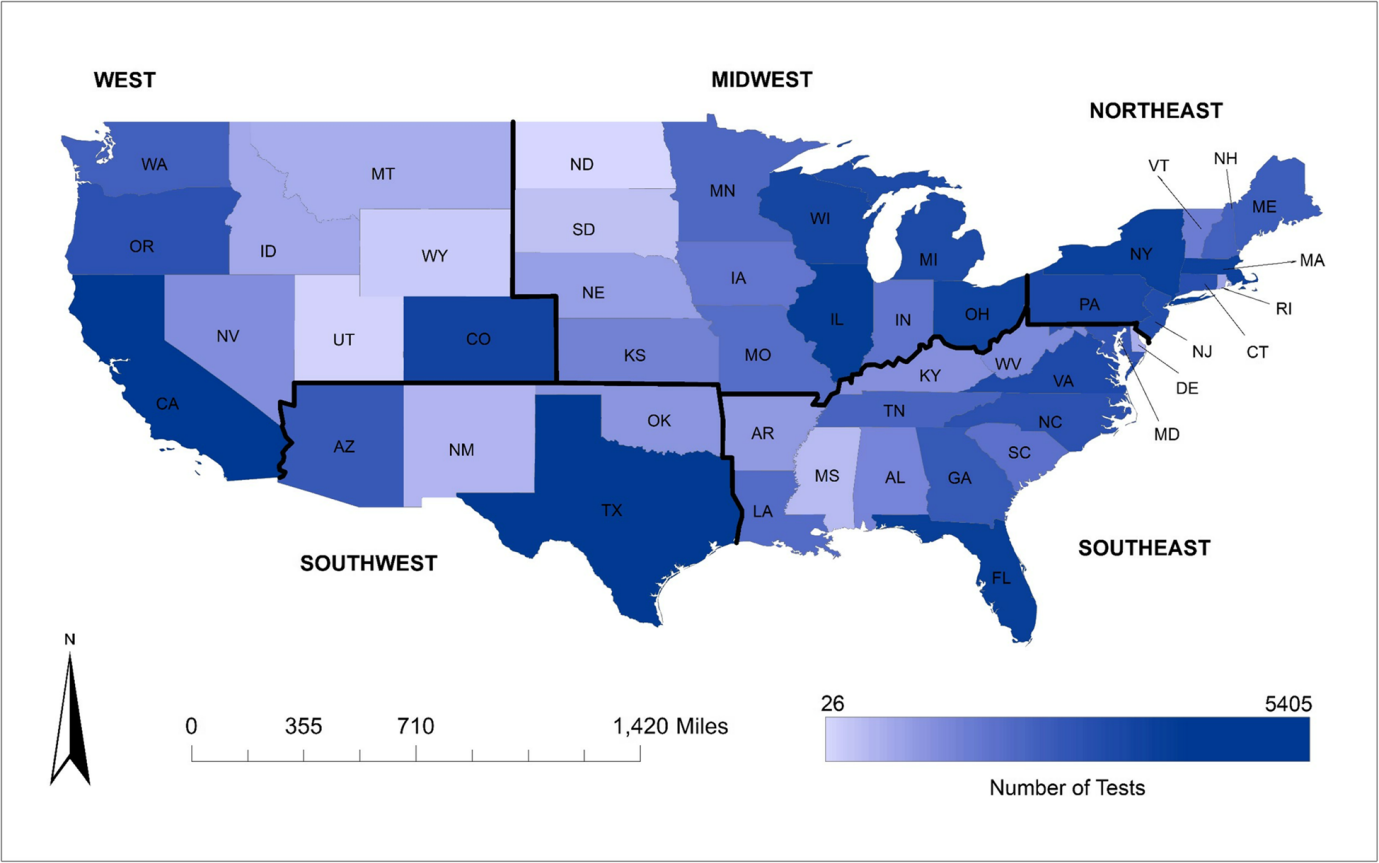
Total number of canine leptospirosis PCR tests for each state across the United States (2009–2016)

The population of dogs tested was 51% female and had a mean age of 6.9 years (SD 0.02; range 0–21). As 38.5% (15,446/40,118) of the entries had breed missing or listed as mixed with no predominant breed designation for the mix, breed group was not examined. PCR test results were available for every state (Fig. 1), with a median of 357 [mean 787; range: 18 (Alaska) - 5405 (California)] test results per state over the study period. At least one PCR test result was available for 44.5% (1401/3143) of counties, with a median of 6 (mean 29; range: 1–1652) tests per county over the study period. Over 18% (262/1401) of counties with test results available only had one test result. A data governance agreement precluded presentation of county-level data in maps.

**Fig. 2:**
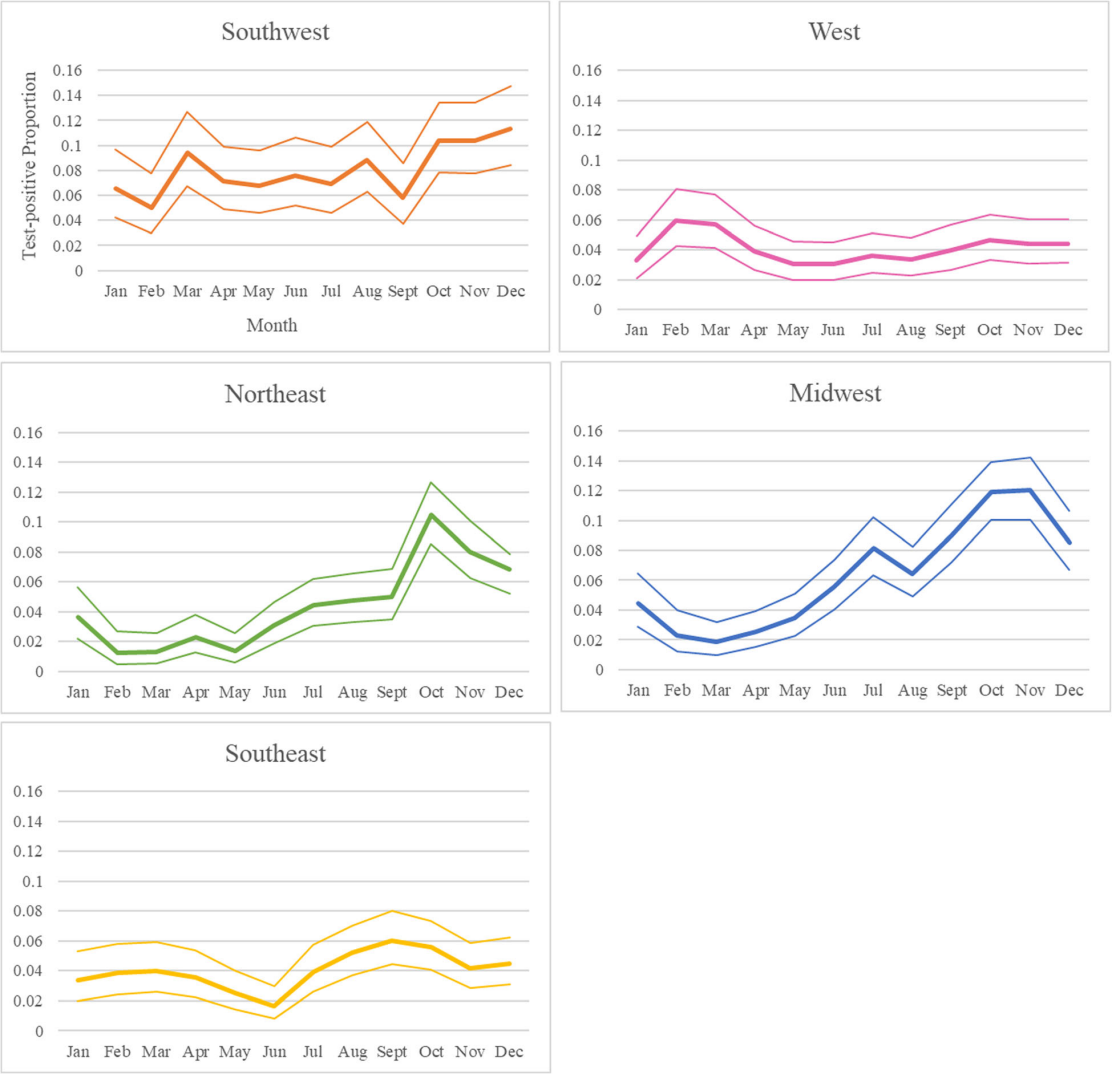
Canine leptospirosis monthly PCR test-positive proportion across the United States by region (2009–2016)

Over the study period, canine leptospirosis PCR test-positive proportion in the United States was 5.4% (2176/40,118) (95% Confidence Interval (95% CI) = 5.2, 5.7%). Test-positive proportion ranged from 2.6% (2010 and 2011) to 8.3% (2013) and varied by month and region. When all years were pooled, the test-positive proportion steadily increased through the spring and summer seasons, and it peaked in the fall in the midwest, and northeast regions (Fig. 2). Test-positive proportion was lowest in February and March in these two regions. Test-positive proportion remained relatively constant in the southeast region, yet it was slightly higher in the fall season, with a dip in June (Fig. 2). Test-positive proportion remained relatively constant across seasons in the west and southwest, with the highest test-positive proportion observed in the winter months (Feb and Dec, respectively) (Fig. 2).

**Fig. 3:**
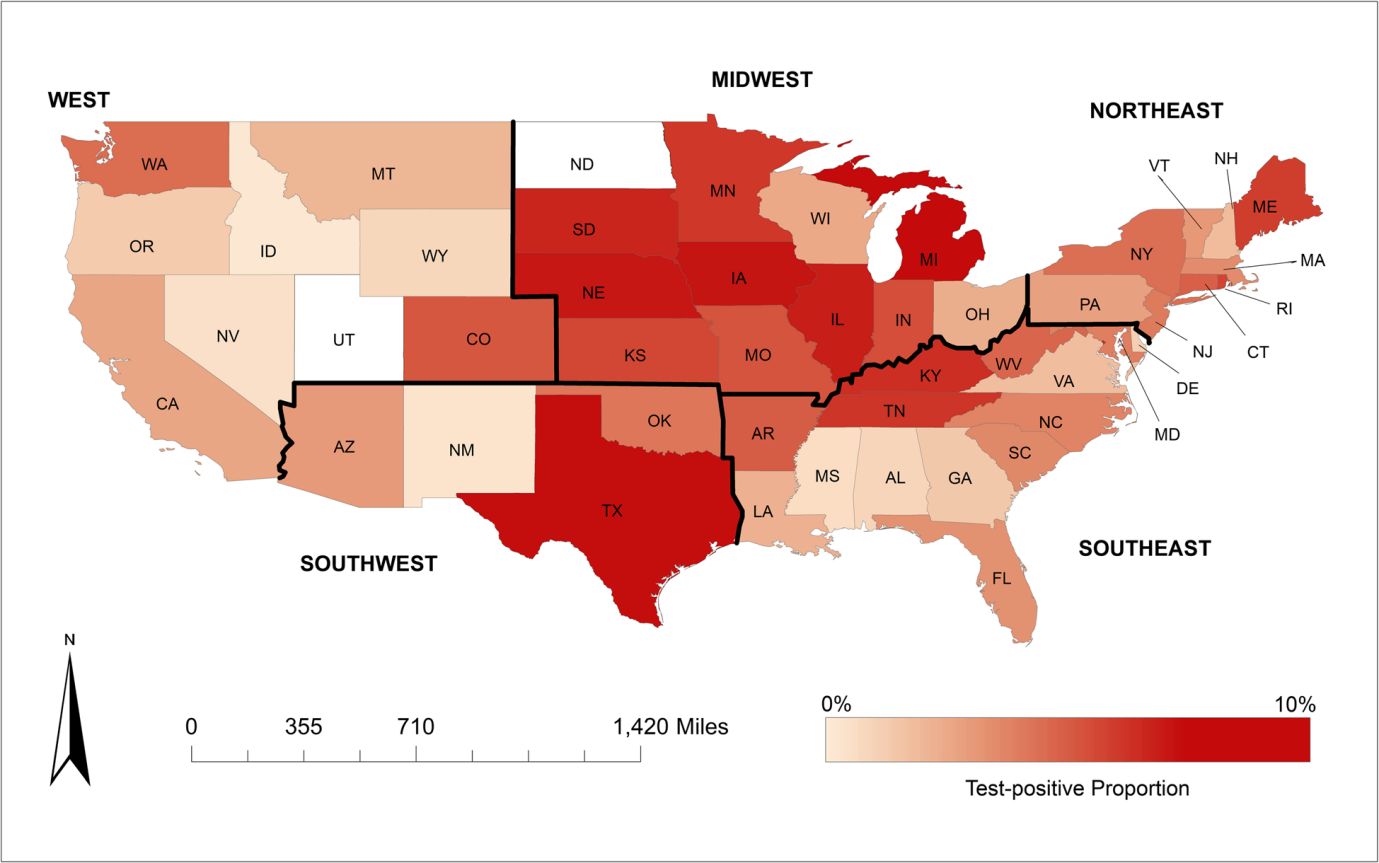
Canine leptospirosis state-level PCR test-positive proportion across the United States (2009–2016)

All states had at least one positive test result except for Utah (total number of tests = 41), North Dakota (26) and Alaska (18) (Fig. 1). Of the counties with at least one test result, 33.9% (475/1401) had at least one positive test result. Test-positive proportion point estimates were highest in the midwest (7.0%) and southwest (8.1%) regions as Michigan (9.6%), Texas (9.1%), Iowa (9.1%), Nebraska (9.0%), and Illinois (8.7%) had the highest state-level test-positive proportion over the study period (Fig. 3, Additional file 1: Figure S1). Test-positive proportion in the other regions was highest for states bordering the midwest and southwest; Kentucky (6.9%) and Colorado (5.8%) had the highest test-positive proportion for their respective regions (Fig. 3, Additional file 1: Figure S1).

### Risk-factor analysis

**Fig. 4:**
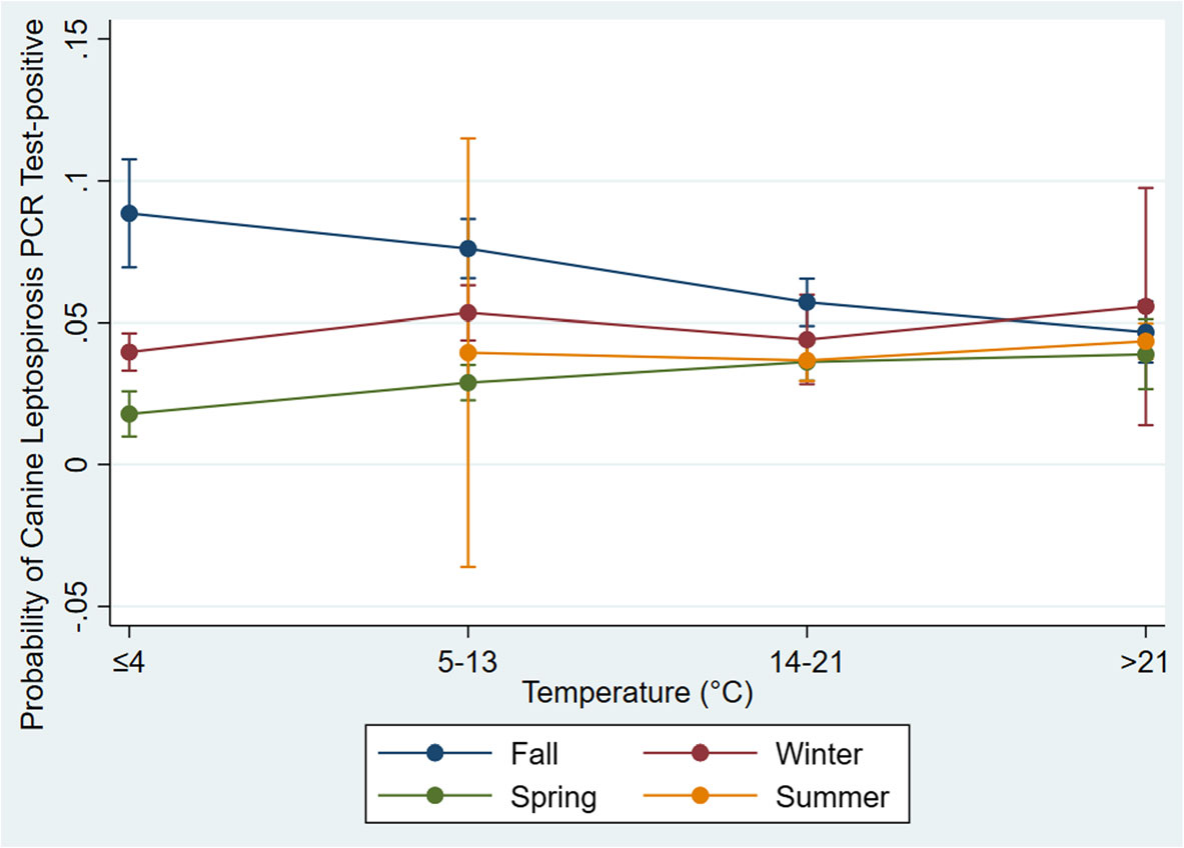
Predictive probability of a positive canine leptospirosis PCR test result for temperature categories by season

**Fig. 5:**
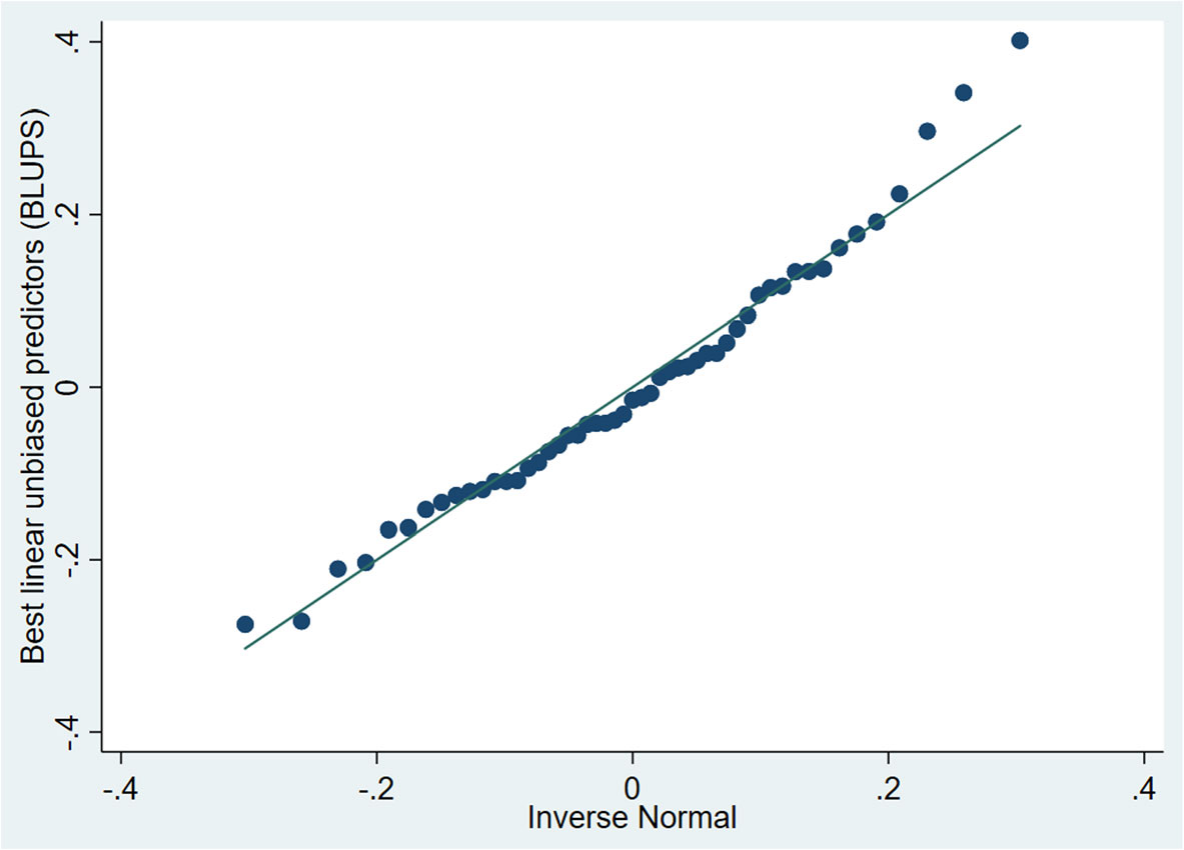
BLUPS for the final multivariable generalized mixed logistic regression model

Of the 13 variables analyzed, 10 were significant predictors of a canine PCR-positive test result in univariable models (Table 1). No variables eligible for the multivariable model were found to be highly correlated. Age, sex, season, year, region, Palmer drought severity index (PDSI), precipitation, and temperature were retained in the final multivariable model as significant predictors for a canine PCR-positive test result (Table 2). Interactions between age and sex, season and PDSI, season and precipitation, and season and temperature were assessed. The interaction terms between season and precipitation (*P* = 0.033) and season and temperature (*P* < 0.001) were significant in the final model. Akaike information criterion (AIC) values were very similar and Bayesian Information Criterion (BIC) value lower for the model with only the season and temperature interaction term [both interaction terms (AIC: 15058.81, BIC: 15444.02); only season and precipitation interaction term (AIC: 15085.44 BIC: 15402.16)]; only season and temperature interaction term (AIC: 15059, BIC: 15367.17); the model with only the season and temperature interaction term was chosen. Predictive probabilities for this model were relatively unchanged across temperature categories for winter, spring and summer seasons, while probabilities decreased with increasing temperatures for the fall season (Fig. 4). The best linear unbiased predictors (BLUPS) followed a normal distribution for this final multivariable model (Fig. 5) suggesting good model fit.

**Table 1:** Univariable mixed logistic regression models for canine leptospirosis PCR tests in the United States (2009–2016)

Variable	N (%)	Odds Ratio (95% CI)	*p*-value
Dog age (years)			< 0.001
0–4	10,612 (27.2)	Reference	
5–7	9503 (24.3)	0.72 (0.64, 0.80)	< 0.001
8–10	10,176 (26.1)	0.49 (0.43, 0.55)	< 0.001
> 10	8752 (22.4)	0.34 (0.30, 0.40)	< 0.001
Dog sex			< 0.001
Female	20,299 (51.3)	Reference	
Male	19,253 (48.7)	1.31 (1.20, 1.43)	
Season test performed			< 0.001
Fall	7604 (18.9)	Reference	
Winter	10,405 (25.9)	0.78 (0.70, 0.88)	< 0.001
Spring	9402 (23.4)	0.47 (0.41, 0.54)	< 0.001
Summer	12,707 (31.7)	0.63 (0.56, 0.72)	< 0.001
Year test performed			< 0.001
2009	4.4% (3.4, 5.6%)^1^	Reference	
2010	2.6% (2.0, 3.2%)^1^	0.57 (0.40, 0.81)	0.001
2011	2.6% (2.0, 3.2%)^1^	0.57 (0.40, 0.80)	0.001
2012	3.8% (3.2, 4.4%)^1^	0.86 (0.63, 1.17)	0.333
2013	7.7% (6.9, 8.5%)^1^	1.81 (1.36, 2.40)	< 0.001
2014	7.5% (6.9, 8.2%)^1^	1.73 (1.31, 2.28)	< 0.001
2015	6.1% (5.6, 6.6%)^1^	1.35 (1.03, 1.78)	0.031
2016	5.0% (4.6, 5.4%)^1^	1.11 (0.84, 1.46)	0.446
County urban influence code^2^			0.013
Non-urban	3337 (8.3)	Reference	
Urban	36,781 (91.7)	1.31 (1.06, 1.61)	
County dog density (dogs/mi^2^)^2^			0.013
0–50	8788 (21.9)	Reference	
51–150	11,059 (27.6)	1.17 (0.98, 1.39)	0.088
151–350	9956 (24.8)	1.41 (1.15, 1.72)	0.001
> 350	10,315 (25.7)	1.32 (1.04, 1.64)	0.020
Region^2^			0.010
Midwest	9536 (23.8)	Reference	
Southwest	4994 (12.5)	0.89 (0.57, 1.40)	0.624
West	9230 (23.0)	0.52 (0.37, 0.75)	< 0.001
Southeast	8032 (20.0)	0.65 (0.48, 0.87)	0.003
Northeast	8326 (20.8)	0.73 (0.53, 0.99)	0.046
State climate division Palmer Drought Severity Index^2,3^			< 0.001
≤ 0 (dry conditions)	20,852 (52.0)	Reference	
> 0 (wet conditions)	19,266 (48.0)	1.24 (1.12, 1.37)	
State climate division precipitation (centimeters)^2,3^			< 0.001
0–2.5	6550 (16.3)	Reference	
2.6–7.6	13,764 (34.3)	1.33 (1.14, 1.56)	< 0.001
7.7–12.7	12,327 (30.7)	1.44 (1.22, 1.70)	< 0.001
> 12.7	7477 (18.6)	1.37 (1.15, 1.64)	0.001
State climate division temperature (°C)^2,3^			0.001
≤ 4	6795 (16.9)	Reference	
5–13	10,460 (26.1)	1.24 (1.07, 1.43)	0.003
14–21	12,550 (31.3)	1.05 (0.91, 1.21)	0.496
> 21	10,313 (25.7)	0.99 (0.85, 1.16)	0.914
County percent of adults with a bachelors degree or higher^2^			0.641
< 50%	36,634 (91.3)	Reference	
≥ 50%	3484 (8.7)	0.97 (0.73, 1.29)	
County median household income (USD)^2^			0.871
< $50,000	6126 (15.3)	Reference	
$50,000–$74,999	23,778 (59.3)	0.97 (0.81, 1.16)	0.731
$75,000–$99,999	8426 (21.0)	0.96 (0.76, 1.21)	0.729
≥ $100,000	1788 (4.5)	1.11 (0.75, 1.66)	0.594
County water cover percentage^2^			0.180
≤ 1%	5929 (14.8)	Reference	
1.01–4%	13,638 (34.0)	1.01 (0.83, 1.23)	0.904
4.01–15%	10,483 (26.1)	1.22 (0.97, 1.53)	0.086
> 15%	10,068 (25.1)	1.13 (0.89, 1.43)	0.334

^1^Test-positive proportion and 95% CI provided, as a data governance agreement precluded inclusion of test numbers by year

^2^Location based on veterinary facility that submitted sample for testing

^3^Climate data for month and year test was performed

**Table 2:** Final multivariable mixed logistic regression model for canine leptospirosis PCR tests in the United States

Variable	Odds Ratio (95% CI)	*p*-value
Dog age (years)		< 0.001
0–4	Reference	
5–7	0.71 (0.63, 0.80)	< 0.001
8–10	0.48 (0.43, 0.55)	< 0.001
> 10	0.35 (0.30, 0.40)	< 0.001
Dog sex		< 0.001
Female	Reference	
Male	1.28 (1.17, 1.40)	
Year test performed		< 0.001
2009	Reference	
2010	0.63 (0.44, 0.90)	0.012
2011	0.65 (0.46, 0.93)	0.018
2012	1.02 (0.73, 1.41)	0.925
2013	2.08 (1.55, 2.80)	< 0.001
2014	1.93 (1.45, 2.57)	< 0.001
2015	1.45 (1.09, 1.93)	0.010
2016	1.22 (0.91, 1.62)	0.178
Region^1^		0.0398
Midwest	Reference	
Southwest	1.05 (0.68, 1.64)	0.816
West	0.67 (0.47, 0.94)	0.022
Southeast	0.68 (0.51, 0.91)	0.009
Northeast	0.80 (0.59, 1.09)	0.165
State climate division Palmer Drought Severity Index^1,2^		< 0.001
≤ 0 (dry conditions)	Reference	
> 0 (wet conditions)	1.24 (1.10, 1.38)	
State climate division precipitation (centimeters)^1,2^		0.0104
0–2.5	Reference	
2.6–7.6	1.30 (1.10, 1.54)	0.002
7.7–12.7	1.32 (1.10, 1.58)	0.003
> 12.7	1.35 (1.10, 1.65)	0.003
Season test performed		< 0.001
Fall	Reference	
Winter	0.41 (0.32, 0.54)	< 0.001
Spring	0.18 (0.11, 0.29)	< 0.001
Summer	0.93 (0.73, 1.18)	0.537
State climate division temperature (°C)^1,2^		< 0.001
≤ 4	Reference	
5–13	0.84 (0.65, 1.08)	0.182
14–21	0.61 (0.47, 0.79)	< 0.001
> 21	0.49 (0.35, 0.68)	< 0.001
Interaction between season test performed and state climate division temperature (°C)^3^		< 0.001

Final model includes data from 2009 through 2016.

^1^Location based on veterinary facility that submitted sample for testing

^2^Climate data for month and year test was performed

^3^Stratum-specific odds ratios not reported

## Discussion

In this study we found the overall canine leptospirosis PCR test-positive proportion to be 5.4% across the United States, between 2009 to 2016. These results indicate the likelihood of a positive versus negative test result, and although likely related to, caution must be used with interpretation of the findings as test positive results do not necessarily correspond with disease status. Previous nationwide canine studies using MAT testing found prevalence to be 4.5–8.1% between 2000 and 2010 [7, 16]. More recently, the MAT positive prevalence for canine leptospirosis in the United States was estimated to be 14.1% between 2000 and 2014 [5]. In our study, fluctuations in test-positive proportion were seen from year to year and ‘year’ was a significant predictor in the final multivariable model. Similar fluctuations in prevalence have been observed by others and multiple factors may be partially responsible [9]. Possible explanations for this are variation in environmental factors or varying veterinarian or dog owner awareness of the disease due to recent outbreaks and media coverage (which likely result in increases in testing, vaccination, and/or animal management changes). In these situations, more tests may be submitted for a wider range of potential cases, and if these are submitted from patients less likely to have leptospirosis, the test-positive proportion could decrease, even if there was an increase in disease prevalence in the overall dog population, since these are independent measures.

Prior studies have identified living in an urban area and poverty status as significant predictors for canine leptospirosis [10–12]. Human sociodemographic variables (education, income) were not significant in our univariable models. Although ‘urban area’ was significant in our univariable model, it did not remain significant in our final multivariable model and was removed. Possible explanations for our differing results are that, in contrast to previous studies, we utilized multivariable analysis, accounted for clustering by county and state, included additional climate variables (e.g., PDSI), and based the model outcome on PCR test results.

High precipitation and wet environmental conditions as designated by the PDSI were identified as significant predictors for a positive leptospirosis test. This is supported by previous studies performed in the United States and Canada, where a high number of canine leptospirosis cases were seen after prolonged periods of rainfall [6, 17]. In the Philippines, human leptospirosis epidemics have been correlated with high rainfall and environmental humidity [18]. As leptospirosis-causing bacteria are often spread through water from the contaminated urine of reservoir species to dogs, it is thought that rainfall and environmental wetness increase transmission opportunities. The interaction between season and temperature was significant and predictive probability plots indicated temperature did not have a large effect on the probability of a dog testing positive during the winter, spring, and summer seasons. In contrast, during the fall season, a dog had the highest probability of testing positive for the lowest temperature category (≤ 4 °C). The probability of a dog testing positive gradually lowered as the temperature increased during the fall season. This finding was surprising as both human and canine leptospirosis has previously been correlated with increasing temperatures, with *Leptospira* found to thrive in temperatures near 30 °C [3, 18, 19]. This discrepancy with our findings may be explained by other factors that impact transmission opportunities, such as the abundance and activity of reservoir hosts and dog activities (i.e. dog park usage) when temperatures drop in the fall. It could also reflect increased consideration of leptospirosis during warmer periods with testing of a wider range of clinical presentations, but more focused testing of higher likelihood clinical presentations in periods less widely associated with leptospirosis risk. In the winter, spring, and summer seasons, the predictive probabilities of a dog testing positive were greatest for the highest temperature category (> 21 °C).

Overall test-positive proportion peaked in the fall months (Sept-Nov). Canine leptospirosis has previously been found to increase in prevalence from late summer into fall [6–8, 12, 19]. In some countries, seasonal trends have been shown to vary by region, perhaps due to varying weather patterns and seasonal canine activities (e.g. swimming in lakes/ponds) across regions [16]. At the state-level, the midwest and southwest regions were observed to have the highest test-positive prevalence of canine leptospirosis. This finding was supported by ‘region’ identified as a significant predictor in the multivariable model, where the odds of testing positive were significantly lower for dogs in the southeast and west compared to dogs in the midwest. These findings align with previous studies that observed clusters of cases and high seroprevalence in similar areas [4, 5]. It is thought that proximity to bodies of water, rainfall patterns, and reservoir host presence could explain the higher prevalence in these areas.

The odds of testing positive in this study were lower for dogs of all age categories compared to young dogs (0–4 years of age). A previous MAT-based study found dogs under a year of age and dogs over 8 years of age to be at increased risk as compared to other age categories [20]. Age findings vary by study, as other studies using MAT found older dogs to be at highest risk of seropositivity or age to not be a significant risk factor [8, 9, 12, 14]. These differences could be due to differing source populations or categorization of dog age. However, younger dogs may be at higher risk due to increased activities and exposures or a lower incidence of effective vaccine coverage. Male dogs had the highest odds of testing positive in this study which is also supported by previous work [9]. This is perhaps due to a more common use for work or sport or other reasons for increased exposure opportunities (e.g. roaming).

Limitations of this study include a lack of confirmatory corresponding clinical data; however, it was assumed that samples were from animals presented to veterinary practices that had clinical disease consistent with leptospirosis. Another limitation was that data were acquired from a single commercial laboratory. As such, some regions of the United States might not have had a centrally located branch of the laboratory and be under-represented in our study. The absence of data from these areas may have influenced the generalizability of our results for the entire United States. Additionally, dog location data were not available, but were surmised from zip code and county data for the sample submitting facility. We expect most dogs lived in the same county of the facility that submitted the sample; however, it is possible that exposure occurred outside of the county of residence due to travel of dogs to other regions. Variations in scale (e.g. variables generalized at the county level) and population-level data (that may not represent the owners of dogs in the study) may have impacted our findings.

Another limitation of the dataset was no unique dog identifier was available. Duplicates may have arisen from the use of universal testing (most commonly encountered from testing involving shelters or dog breeders), or repeated testing for a given dog. We expect this to have had minimal impact on this study as duplicates were uncommon (3% of total data) and were subsequently removed. Additionally, a very small percentage (0.2% of total data) of these duplicates were discordant results. Although testing instructions suggested paired blood and urine samples be submitted for a given dog, it was unknown if both or only one of the sample types were submitted for each dog. It is possible duplicate entries arose from paired blood and urine samples from a single dog. Following acute infection, leptospires are present in the blood the first 10 days of infection [21]. Following this bacteremic phase, leptospires are then shed in the urine [3]. Therefore, blood is the sample of choice in the first 10 days of illness, and then urine is the sample of choice after the first week of illness. However, the timeline of infection is often unknown and testing both blood and urine may increase diagnostic sensitivity. As sample type was unknown, the samples submitted could have thereby altered the test sensitivity and our reported test-positive proportion and risk factors. Additionally, commercially available PCR assays are not able to determine the infecting serovar or serogroup for a positive test result [3]. This is a limitation, as serovar identification is important to further understand the serovar-specific epidemiology of this disease (e.g. identify reservoir species involved in transmission to canines) and inform prevention strategies (e.g. are observed serovars included in the current vaccine).

## Conclusion

We observed positive canine leptospirosis PCR tests to be widespread across the United States. Our risk factor analysis identified dog demographic (age, sex), spatio-temporal (region, year), seasonal (season), and environmental (precipitation, PDSI, temperature) variables as significant predictors of a positive canine leptospirosis PCR test. These findings can be used to target leptospirosis prevention practices at clients with dogs at increased risk, potentially positively influencing both dog and owner health. Research collecting additional dog-level data (e.g. home location, dog owner sociodemographic variables, vaccination and exposure histories, husbandry practices) is needed to further investigate canine leptospirosis and identify effective prevention strategies.

## Methods

### Data acquisition

Data were obtained from canine leptospirosis RealPCR tests from IDEXX Laboratories, Inc. submitted by United States veterinarians from January 1, 2009 to December 31, 2016. Tests were conducted on blood and/or urine samples from canine patients. Data from IDEXX Laboratories, Inc. included sample submitting location (e.g., veterinary facility state, county, zip code), test date, dog demographics (breed, sex, date of birth), and test outcome (positive or negative). Permission to access and use data was obtained from IDEXX Laboratories, Inc. From these data, variables were derived for the dog’s age at the time of testing, year and season of when testing was performed (Fall: September–November, Winter: December–February, Spring: March–May, Summer: June–August); and region (west, midwest, northeast, southeast, southwest) of the United States based on the state from which the sample was submitted (Fig. 1). Alaska and Hawaii were included in the West region. Duplicate entries were removed; due to the lack of unique identifiers, an entry was considered a duplicate if more than one entry had the same dog date of birth, zip code, breed, and sex, and the test dates in which each test was performed were within 7 days of each other. If the test outcomes for a set of duplicate entries were the same, the most recent entry was retained in the dataset and additional entries were removed. If the test outcomes differed for a set of duplicates, all entries were removed. This resulted in a dataset to best capture dog-level data for illness events for the period.

Publicly available data were used to derive additional environmental and socioeconomic variables. The 2013 county urban influence codes were used to designate a county as urban or non-urban. Socioeconomic variables included county annual human population estimates, county median 2016 household income, and county average percent of adults with a bachelor’s degree or higher for 2012–2016. The annual human population estimates were used in conjunction with the American Veterinary Medical Association’s dog population formula to estimate the number of owned dogs residing in each county [22]. Dog density for each county was calculated by dividing the estimated county owned dog population by county area (square miles). County water cover percentage was calculated by dividing water area (square miles) in each county by overall county area (square miles). Precipitation, temperature, and PDSI were obtained for each month over the study period by state climate divisions. Palmer Drought Severity Index is a long-term measure of environmental dryness based on temperature and precipitation that ranges from − 10 (drought) to 10 (moist) [23].

### Data analysis

Test-positive proportion of leptospirosis in the United States at the dog-level was calculated overall, for spatial subgroups (for each state and county) and for each year by dividing number of positive canine leptospirosis PCR tests by total number of tests. Ninety-five percent Clopper-Pearson confidence intervals were also calculated. Test-positive proportion was mapped by state and county for all years combined (2009 through 2016) using ArcGIS version 10.2.2 (Environmental Systems Research Institute).

The association between thirteen independent variables and a positive leptospirosis PCR test was explored using generalized linear mixed logistic regression models that accounted for the clustering of tests within county and state to control for correlations within counties of the same state. The main outcome of interest was a positive canine leptospirosis PCR test. Descriptive statistics, OR and 95% CI for the ORs were calculated for all variables. For model building; first, linearity was assessed between all continuous variables and the outcome using logit transformed linear smooth plots to meet assumptions of the generalized linear model. When the linearity assumption was not met, variables were dichotomized by median or categorized into quartiles: age (0–4 years, 5–7, 8–10, > 10), season (Fall: September/October/November, Winter: December/January/February, Spring: March/April/May, Summer: June/July/August), precipitation (≤2.54 cm, 2.55–7.62, 7.63–12.70, > 12.70), temperature (≤4 °C, 5–13, 14–21, > 21), PDSI (≤0, > 0), dog density (≤50 dogs/mi^2^, 51–150, 151–350, > 350), percentage of adults with a college education (bachelor’s degree or higher) (< 50%, ≥50%), median household income (<$50,000 USD, $50,000–$74,999, $75,000–$99,999, ≥$100,000), and percentage of water cover (≤1.00%, 1.01–4.00%, 4.01–15.00, > 15.00%).

Univariable generalized mixed logistic regression models using county within state as random effects were built and variables with a likelihood ratio test *p*-value < 0.2 were eligible to be tested for inclusion in the final multivariable model. Spearman’s rank correlation was performed between all predictors eligible for multivariable analysis. When predictors were highly correlated (correlation coefficient ≥ |0.80|), one variable was retained based on perceived importance. A final multivariable generalized mixed logistic regression model accounting for clustering of tests within county and state was built using a backwards stepwise approach. Confounding was assessed when removing variables from the multivariable model. Variables were kept in the model as confounders if their removal changed the coefficients of one or more retained variables by ≥20% and the variable was related to both the retained variable(s) and outcome (assessed using a directed acyclic graph). Statistical significance was declared at a likelihood ratio test *p*-value < 0.05. Plausible 2-way interactions between variables retained in the final multivariable model were assessed for significance using a likelihood ratio test. The AIC and BIC were used to assess the fitness of the models and compare final multivariable models with and without the interaction term(s). The model with lower values and best fit was selected. Predictive probabilities and associated 95% CIs for a positive test result were graphed to visualize interaction terms. BLUPS for the selected final multivariable model were estimated and assessed for normality. Stata 15 (StataCorp, College Station TX) was used for analysis.

## Supplementary information

**Additional File 1: Figure S1.** Canine leptospirosis PCR test-positive proportion and 95% confidence intervals by state for the United States (2009–2016)

## Data Availability

PCR test result data that support the findings of this study are available from IDEXX Laboratories, Inc. but restrictions apply to the availability of these data, which were used under agreement for the current study, and so are not publicly available. Data are however available from the authors upon reasonable request and with permission of IDEXX Laboratories, Inc. The remainder data that support the findings of this study are publicly available online at https://www.ers.usda.gov/data-products/county-level-data-sets/download-data/, https://www.census.gov/geographies/reference-files/time-series/geo/gazetteer-files.2010.html and https://www.ncei.noaa.gov/data/global-summary-of-the-month/.

## Abbreviations

95% CI: 95% confidence interval
AIC: Akaike Information Criterion
BIC: Bayesian Information Criterion
BLUPS: Best linear unbiased predictors
MAT: Microscopic agglutination test
PCR: Polymerase chain reaction
PDSI: Palmer drought severity index
