# Supplementary material for: A cross-sectional study of environmental, dog, and human-related risk factors for positive canine leptospirosis PCR test results in the United States, 2009 to 2016
Source: BMC Vet Res. 2019 Nov 15;15:412. doi: 10.1186/s12917-019-2148-6 (PMC6858729; doi:10.1186/s12917-019-2148-6)
Supplement: Supplementary file 1 — Additional File 1: Figure S1. Canine leptospirosis PCR test-positive proportion and 95% confidence intervals by state for the United States (2009–2016) [file 12917_2019_2148_MOESM1_ESM.docx]

| **State** | **Prevalence (%)** | **95% Confidence Interval (%)** |
| --- | --- | --- |
| Alabama | 1.5 | 0.3, 4.4 |
| Alaska | 0 | 0, 18.5 * |
| Arizona | 4.1 | 2.8, 5.9 |
| Arkansas | 5.7 | 2.6, 10.5 |
| California | 4.0 | 3.5, 4.6 |
| Colorado | 5.8 | 4.8, 7.1 |
| Connecticut | 5.6 | 4.3, 7.3 |
| Delaware | 2.1 | 0.3, 7.3 |
| District of Columbia | 7.1 | 2.4, 15.9 |
| Florida | 4.2 | 3.4, 5.1 |
| Georgia | 2.6 | 1.6, 4.0 |
| Hawaii | 2.7 | 1.0, 5.7 |
| Idaho | 0.7 | 0.02, 4.3 |
| Illinois | 8.7 | 7.9, 9.8 |
| Indiana | 6.0 | 3.6, 9.3 |
| Iowa | 9.1 | 5.9, 13.2 |
| Kansas | 6.0 | 3.1, 10.2 |
| Kentucky | 6.9 | 3.6, 11.8 |
| Louisiana | 3.6 | 2.0, 5.9 |
| Maine | 6.4 | 4.6, 8.6 |
| Maryland | 4.7 | 3.3, 6.4 |
| Massachusetts | 4.3 | 3.4, 5.4 |
| Michigan | 9.6 | 7.9, 11.6 |
| Minnesota | 6.4 | 4.4, 9.1 |
| Mississippi | 1.1 | 0.03, 6.2 |
| Missouri | 5.9 | 3.7, 8.9 |
| Montana | 3.4 | 1.0, 8.5 |
| Nebraska | 9.0 | 5.0, 14.7 |
| Nevada | 1.1 | 0.1, 4.0 |
| New Hampshire | 3.4 | 2.0, 5.3 |
| New Jersey | 4.7 | 3.5, 6.2 |
| New Mexico | 1.1 | 0.02, 5.7 |
| New York | 5.1 | 4.1, 6.2 |
| North Carolina | 4.6 | 3.4, 6.1 |
| North Dakota | 0 | 0, 13.2 * |
| Ohio | 3.7 | 2.9, 4.7 |
| Oklahoma | 4.8 | 2.1, 9.3 |
| Oregon | 2.3 | 1.4, 3.6 |
| Pennsylvania | 4.0 | 3.0, 5.4 |
| Rhode Island | 6.0 | 2.6, 11.5 |
| South Carolina | 4.5 | 2.5, 7.2 |
| South Dakota | 8.1 | 3.0, 16.8 |
| Tennessee | 6.5 | 4.4, 9.1 |
| Texas | 9.1 | 8.2, 10.0 |
| Utah | 0 | 0, 8.6 * |
| Vermont | 4.2 | 1.9, 7.8 |
| Virginia | 2.9 | 2.0, 4.2 |
| Washington | 5.1 | 3.5, 7.2 |
| West Virginia | 5.2 | 2.5, 9.3 |
| Wisconsin | 3.9 | 2.9, 5.2 |
| Wyoming | 1.5 | 0.04, 8.0 |
| **One-sided, 97.5% confidence interval* | | |
